# The Molecular Landscape of Colorectal Laterally Spreading Tumors: From Endoscopic Subtypes to Molecular Targets

**DOI:** 10.3390/ijms26178445

**Published:** 2025-08-30

**Authors:** Mara Martinelli, Nicoletta Cascelli, Ottavia Bartolo, Mario Ciuffi, Carmela Mazzoccoli, Rosalia Dieli, Rosa Lioy, Matteo Landriscina, Carlo Calabrese, Fabiana Crispo

**Affiliations:** 1 Laboratory of Preclinical and Translational Research, IRCCS-CROB, Referral Cancer Center of Basilicata, 85028 Rionero in Vulture, Italy; mara.martinelli@crob.it (M.M.); nicoletta.cascelli@crob.it (N.C.); rosalia.dieli@crob.it (R.D.); rosa.lioy@crob.it (R.L.); carlo.calabrese@crob.it (C.C.); 2 Endoscopy Unit, IRCCS-CROB, Referral Cancer Center of Basilicata, 85028 Rionero in Vulture, Italy; mario.ciuffi@crob.it; 3 Hospital Pharmacy, IRCCS-CROB, Referral Cancer Center of Basilicata, 85028 Rionero in Vulture, Italy; carmela.mazzoccoli@crob.it; 4 Unit of Medical Oncology and Biomolecular Therapy, Policlinico Riuniti, 71122 Foggia, Italy; matteo.landriscina@unifg.it; 5 Department of Medical and Surgical Sciences, University of Foggia, 71122 Foggia, Italy

**Keywords:** lateral spreading tumors, colorectal cancer, inflammatory bowel diseases, genetic profile, epigenetic features

## Abstract

Lateral Spreading Tumors (LSTs) are a type of non-polypoid lesion known for their flat morphology, which often leads to them going undetected. However, especially nongranular (NG) LSTs have the potential for malignant transformation. Recent advances in endoscopic technologies have improved the detection of these lesions. Despite growing research interest in their role in colorectal cancer (CRC) development, a comprehensive molecular characterization of LSTs is still lacking. The aim of this review is to highlight the current knowledge of the molecular characteristics of LSTs, that may help in determining whether LSTs can be prognostic indicators and identifying cases where they may rapidly progress to CRC through characteristic molecular pathways. From a mutational point of view, LSTs seem to be more closely associated with inflammatory bowel diseases (IBDs) than with polypoid lesions. Nonetheless, they have peculiar epigenetic and genetic traits, which set them apart from other adenomas or bowel diseases. Elucidating their role in CRC development would provide benefits for their classification and management, by enhancing clinical surveillance strategies for patients diagnosed with these lesions in order to improve the efficient prevention of colorectal cancer.

## 1. Introduction

Colorectal cancer (CRC) ranks among the top three most frequently diagnosed cancers worldwide and the second most fatal cancer globally, thus it becomes a substantial public health challenge [1,2]. CRC is a heterogeneous disease, caused by the stepwise progression of gradual genetic and epigenetic aberrations accumulated in oncogenes and tumor suppressor genes [3]. Such changes may occur in two types of precancerous formations, polypoid adenomas and non-polypoid lesions [4]. Adenomatous polyps (adenoma and serrated polyps) are glandular tumors, exhibiting as either low- or high-grade, and accounting for 90% of CRC cases [5]. They are considered precursors of CRC, leading to tumor development through the well-documented multistep “genetic adenoma–carcinoma sequence” [3,6]. Indeed, the patients with these lesions are the main protagonists of current screening, surveillance, and prevention actions. The widespread use of colonoscopy has led to a decrease in both the incidence and mortality rates of CRC by identifying and removing these early-stage precancerous lesions [7].

On the other hand, non-polypoid lesions display distinct characteristics, often spreading to deeper layers at an early stage. While the carcinogenesis of these lesions remains controversial, some evidence suggests greater invasive capabilities to penetrate submucosal layers [8,9]. Within the spectrum of flat colorectal lesions, laterally spreading tumors (LSTs) have been reported as peculiar precursors of CRC. LSTs are characterized by a broad, flat, sideward growth morphology [10,11,12,13]. The knowledge about these lesions is limited and sometimes uncertain, largely due to the lower abundance of reported cases [14]. Patients are typically asymptomatic, leading to frequent mistaken oversight. Furthermore, these lesions are often under-recognized: their flat morphology hinders the detection by common optical colonoscopies that rely on seeing abnormal tissue directly. Thus, lesions need to be a certain size to be spotted during endoscopic investigation.

The incidence of LSTs in routine colonoscopy is only around 9%, probably because of their flat morphology, smooth surface, indistinct boundaries or being isochromatic compared to the background that makes these lesions easy to miss. Moreover, when detected, these infrequent lesions were also overlooked as precursors to CRC, because they were perceived as benign and less invasive compared to polypoid lesions [15]. Consequently, LSTs are often un-resected, allowing them to progress to malignancy within a short span of time [16]. Instead, benign LSTs can develop into colorectal adenocarcinoma within 3 years [17] with a probability from 8.4% to 52.5% [18] on the basis of their molecular characteristics.

**Figure 1:**
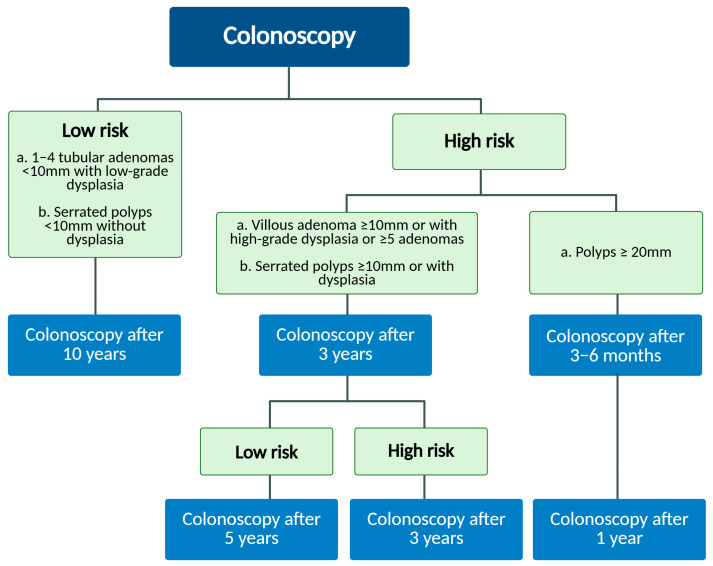
Schematic diagram of recommended post-polypectomy endoscopic surveillance timeline, according to ESGE guidelines [21].

In recent years, considerable advancements in endoscopy technologies, as well as a greater comprehension of the physiopathology features of LSTs, have facilitated the better detection and treatment of these hard-to-spot lesions, enhancing outcomes for CRC patients [19,20]. Moreover, the increased interest of the research community about LST molecular characterization is increasing since they appear to be closely linked to CRC development. Thus, the early detection of potentially precancerous flat lesions might decrease the incidence of colorectal tumors. The staging and clinical decision-making regarding the management and follow-up of patients with LST diagnosis remain subjects of debate, due to the limited understanding of the molecular features that could reveal potential prognostic indicators for CRC onset. Following the European Society Gastrointestinal Endoscopy (ESGE) guidelines, endoscopy surveillance post-polypectomy is applied after 10 years for low-risk patients, and after 3 years for high-risk ones. After 3 or 5 years, a second surveillance colonoscopy is recommended for the high-risk group if high-risk adenomas are further detected, or not, respectively (Figure 1).

According to those dichotomization criteria, if detected, patients who have LSTs belong to the high-risk group. Indeed, researchers have speculated that the progression of LSTs to CRC may be even faster than the other flat lesions and require a series of processes similarly to the adenoma–carcinoma sequence, regulated by the aberrant activation of a variety of proto-oncogenes and inactivation of tumor suppressors and apoptosis-related genes. Nevertheless, an updated molecular characterization, elucidating the process and mechanisms of LST transformation, could help to comprehend if and how faster high-risk LSTs can evolve to cancer, on the basis of their genetic/epigenetic alterations [22]. Finally, in clinical practice, the molecular characterization of LSTs may support clinicians in planning the sanitary surveillance of patients with LSTs. In this perspective, the present review is thought to examine the latest knowledge concerning the molecular features of LST lesions, particularly those that may provide insights about their progression towards malignancy, in order to improve colorectal tumors’ early-stage diagnosis.

## 2. Morphological Characteristics of Laterally Spreading Tumors and Risk of Submucosal Invasion

**Figure 2:**
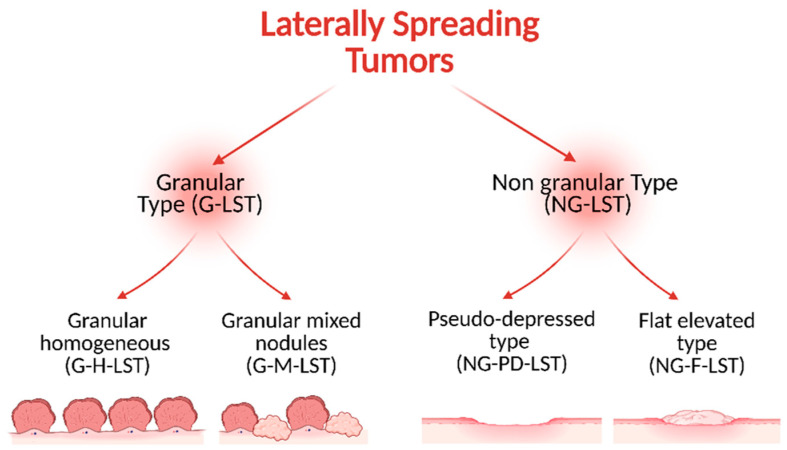
Representative image of LSTs’ classification according to Kudo et al. [23]. This system divides LSTs into two principal categories based on their morphological characteristics. Granular types (G-LSTs) are further classified in the homogeneous subtype (G-H_LST), with a uniform granular surface without nodular components, and mixed-nodular subtype, characterized by a heterogeneous morphology with nodular areas interspersed within the granular surface. Nongranular flat lesions (NG-LSTs) may be distinct in pseudo-depressed (NG-PD-LST), consisting of a central depression surrounded by slightly elevated margins, and flat-elevated subtypes (NG-F-LST), peculiar to a smooth, elevated surface with minimal or absent granularity.

In 1993, Kudo defined the Laterally Spreading Tumors as lesions with a diameter greater than 10 mm and a low vertical axis extended laterally along the luminal wall [23]. According to Kudo’s classification, the LSTs are usually classified into two subtypes depending on the endoscopic findings: granular type (G-LST), with granules and nodules on the tumor surface, and nongranular type (NG-LST) with a flat, smooth surface (Figure 2).

G-LSTs can be further categorized into 2 subgroups: G-H-LST (homogeneous type) with granules of the same size and shape smaller than 3 mm diameters; and G-M-LST (mixed nodules) with granules and nodules on the tumor surface. The NG-LSTs can also be sub-divided into two groups: NG-PD-LST (pseudo-depressed type) with a badly delineated, basin-like depression in the tumor center, and NG-F-LST (flat-elevated type) without depression [24]. Many LSTs are localized in the proximal colon [18] where the homogeneous granular and flat elevated nongranular LSTs are mostly observed, while nodular mixed granular and pseudo-depressed nongranular LSTs are more evenly distributed over the colon [13].

Similarly, the Paris Endoscopic Classification of Superficial Neoplastic Lesions refers to the entire gastrointestinal tract, but its application to the colon is limited in comparison with Kudo’s classification. This classification system assesses the height of the LSTs relative to the closed cups of biopsy forceps (5 mm). LSTs that extend beyond this limit are classified as protruded, whereas those that do not are classified as flat [25]. The application of the endoscopic LST classification is the first step to determine the risk of submucosal invasion.

Even if not necessarily all LSTs progress to cancer, many studies demonstrated their invasive capability through the risk of submucosal invasion (SMI), which depends on morphology, location, and size of the lesions. In a prospective multicenter study, it was observed that invasion occurs more frequently in NG-LSTs than in G-LSTs, with rates of 15.3% and 3.2% of cases, respectively [26,27]. Furthermore, through a meta-analysis of 2949 compared studies, a different risk of SMI among the LST subtypes was observed: 0.5% for G-H-LST against 10.5% for G-M-LST; 31.6% for NG-PD-LST against 4.9% for NG-F-LST. Moreover, SMI risk is more common in distally rather than in proximally located LSTs and it increases as the size of the lesion increases [28].

## 3. Genetic and Molecular Features of LSTs

The study of genetic and transcriptional changes in the progression of LSTs lagged behind the current insight into polypoid lesions. The molecular characteristics of CRC are well known as well as the molecular pathways, such as chromosomal instability (CIN) and microsatellite instability (MSI), which support the progression from adenomas to adenocarcinoma [29]. Many colorectal cancer cases exhibit the mutation or deletion of tumor protein p53 (*TP53*) and adenomatous polyposis coli (*APC*), furthermore, they are supported by gain-of-function mutation in the catenin beta 1 (*CTNNB1*) gene which leads to the constitutive Wnt signaling activation, a pivotal event in the initiation of adenomas with protruding appearance (polypoid) [30,31].

However, the current knowledge about molecular features of precursor lesions allows to distinguish the pathogenesis driver of colorectal cancer, between the adenoma–carcinoma pathway and the serrated pathway, resulting in a lack of detailed molecular characteristic information [32,33], useful for an effective preventive approach and new treatment strategies for subsets of patients. Initial genetic comparisons between non-polypoid and polypoid neoplasms date back to 1994; since then, the investigation of genetic features of LSTs in light of their possible malignant evolution have been ongoing [34,35,36].

Activating the Kirsten rat sarcoma virus (*KRAS*) mutation status is the most widely studied molecular feature for LSTs, as well as adenomatous polyps, because it regulates several pathways related to cell proliferation and survival [37]. In CRCs, *KRAS* is frequently mutated in the early stage of tumor development, particularly in adenomas with high-grade dysplasia [38,39,40,41]. Data are heterogeneous and sometimes contradictory, as highlighted by a meta-analysis conducted by Voorham and his collaborator [42], so it is difficult to have a clear knowledge of KRAS mutation frequency in LSTs. Generally, *KRAS* mutations are more commonly found in G-LSTs compared to NG-LSTs [43], suggesting a potential link between *KRAS* mutations and the frequent occurrence of polypoid foci in granular types. In light of this, *KRAS* mutation status may serve as a reliable biomarker for assessing cancer risk in LSTs. Indeed, if G-LSTs show a mutation burden like polypoid counterparts, their malignant potential may probably arise in time with a mutational analysis on biopsy samples. Moreover, lesions with granular components seem to share a closer genetic resemblance to malignant lesions, with respect to NG-LSTs. However, NG-LSTs are clinically characterized as more aggressive, particularly the pseudo-depressed type, with a higher incidence of advanced carcinoma due to increased abilities in submucosal invasion [44]. They display a more nuclear accumulation of β-catenin and high expression of *MYC*, as well as distinctiveness of mucin 5 (MUC5) expression, indicating the specific features of a morphologically distinct tumor [45].

It is intriguing that the synergistic effect among *KRAS* mutation, cyclooxygenase-2 (COX-2), and gastrin, in promoting colorectal tumorigenesis from LSTs [46,47]. COX-2 facilitates cell growth and inflammatory responses [48,49,50], while gastrin stimulates histamine synthesis and acts as a growth factor for normal gastric mucosa and malignant stomach tumors [51]. Studies have explored the correlation between *KRAS* mutation and COX-2 overexpression in colorectal tumors [52] and colon cancer cell lines [53], revealing that the activation of mutated *KRAS* increases COX-2 expression by enhancing the activity of mitogen-activated protein kinases (MAPKs). This relationship is observed in colorectal adenomas [52] as well as in LSTs, especially G-LSTs. Gastrin expression is higher in G-LSTs compared to NG-LSTs, resembling the genetic alterations observed in polypoid-type colorectal tumors [54].

Conversely to *KRAS*, the loss of the *APC* gene, located on chromosome 5 and correlated with the nuclear localization of oncoprotein β-catenin [54], is more frequent in NG-LSTs rather than G-LSTs [22,45], while several studies show a lower frequency of *APC* mutations in LSTs compared to polypoid lesions, suggesting a different way undertaken by flat-type adenomas for neoplastic transformation.

APC regulates cell growth, and prevents tumor formation by modulating the Wnt signaling pathway, whose activation leads to the transcription of genes involved in tumorigenesis such as cyclin D1 (*CCND1*) and c-*MYC*. Moreover, APC sustains the proteasomal degradation of cytoplasmic β-catenin, preventing its nuclear translocation [55]. Thus, loss-of-function by deletion or protein inactivation by mutation causes the aberrant activation of Wnt signaling and tumorigenesis due to elevated β-catenin levels and the over-expression of its downstream target genes [56,57,58]. The alteration of the Wnt/β-catenin pathway through 5q Loss of Heterozygosity (LOH) is primarily associated with the development of NG-LSTs, while the increased occurrence of *APC* mutations in malignant lesions suggests that this genetic event occurs during carcinomatous evolution. These distinct mechanisms of *APC* disruption contribute to variations in the lesion phenotype of the flat-type or polypoid-type, with different impacts on crucial molecular processes for colorectal cancer onset [59]. Lu et al. recently proposed a novel pathway for LST development, suggesting that *APC* truncation acts as a proto-oncogenic driving mutation leading to Golgi fragmentation, reduction in anoctamin 5 (ANO5) expression, increase in ataxia telangiectasia mutated (ATM) expression, and stabilization of p53 mutated forms. All these alterations affect structural protein reorganization, contributing to CRC development [60]. Notably, the coexistence of the loss of chromosome 5q and a low incidence of *APC* mutations is also found in ulcerative colitis-associated CRS, suggesting similarities in the origin and progression of colitis-associated CRS with LSTs [61].

In addition to *APC* disruption, other alternative mechanisms may influence the Wnt pathway dysregulation in LSTs and include the following: the hypermethylation of the *APC* promoter [62]; miRNA regulators of the Wnt pathway [63]; β-catenin mutations [64]. In LSTs, *CTNNB1* mutations are observed mainly in NG-LSTs, albeit at lower levels compared to protruded intramucosal and advanced CRCs [22,65,66]. Investigating molecular markers for cell polarity and basement membrane components to explain NG-LSTs’ ability to spread laterally [67], Wang et al. showed that adherence proteins like E-cadherin, tetraspanin-5 (TM4SF9), and galectin-1 are lacking in LST cell lines, attributed to their distinctive lateral, superficial growth [68].

The first piece of evidence of a comparable malignant potential of LSTs to their polypoid counterparts regards *TP53* gene status. Located on chromosome 17p, *TP53* is vital for repairing cellular damage and maintaining genomic stability, triggering apoptosis, preventing mutation accumulation, and carcinogenesis [69,70,71]. *TP53* mutations predominantly occurred in cancer samples with the intramucosal invasion of the NG-LST type (74% vs. 33% of G-LST) [72]. Sakai et al. demonstrated that *TP53* mutations increase in cancer cells from adenomas, suggesting independent contribution to malignant transformation in LSTs regardless of subtype [66]. Beyond mutation events, Orita et al. reported that loss of heterozygosity at 17p is the predominant event (97% occurrence) in early-stage flat-type tumors [73]. Nevertheless, loss of p53 appears crucial in LST progression to cancer, but not in submucosal invasion.

Despite being less frequently reported, alterations in genes associated with the transforming growth factor beta (TGFβ) and phosphoinositide 3-kinase (PI3K) pathways are both recognized for LST transformation in colorectal cancer, while *BRAF* mutation is rarely detected in LSTs [74,75,76]. Phosphatidylinositol-4,5-bisphosphate 3-kinase catalytic subunit alpha (*PIK3CA*) mutations, associated with *KRAS* foster cell growth in various cancers, including CRC [77,78]. However, there are some controversial results regarding *PIK3CA* mutations in LSTs. Kaji et al. showed its mutations exclusively in higher-grade LSTs [43], while Konda reported a high frequency of *PIK3CA* alterations in large LST-G, mainly in high-grade dysplasia or T1 cancer histological status [79].

Few studies have explored axon guidance molecules in LSTs. Notable mentions include *SLITRK1*, *SLITRK2*, *SLIT1*, and *NTRK1*, *NTRK2*, and *NTRK3* genes, which exhibit recurrent mutations and epigenetic inactivation in LSTs [80]. This aspect resembles the mutational pattern observed in CRC development. Hesson et al. identified key genetic mutations in LSTs associated with their cancer evolution, involving signaling pathways related to axonal guidance, Wnt, and the actin cytoskeleton [80]. Axonal guidance genes and their receptors play vital roles in axonal development and cell morphogenesis across various tissues. These pathways impact carcinogenesis by influencing processes such as angiogenesis, cell survival, apoptosis, and cell positioning/migration. Multiple alterations in key axonal guidance molecules such as semaphorins [81], netrins [82,83], and ephrins [84], are frequently observed in CRC since they are involved in tumorigenesis [81,82,84] and are also implicated in LSTs [85]. For instance, SLIT2, an inhibitor of leukocyte chemotaxis and involved in neutrin interactions mediating apoptotic pathways, is frequently inactivated in CRC by hypermethylation of the promoter region [86]. Moreover, mutations in mediator complex subunit 12L (*MED12L*), a transcriptional coactivator, were identified in LSTs. This protein acts as an oncogene in colorectal cancer, and its reduced expression leads to a decrease in cancer cell proliferation [87]. Specific features associated with LSTs, known as “skirts,” have sometimes been detected. Skirts are slightly elevated flat lesions with wide pits spreading across the margins of LSTs, more commonly found in the rectum than in the colon. They often occur near mixed LSTs of the granular-nodular mixed type and exhibit characteristics resembling low-grade dysplasia, like hyperplastic polyps [88]. The genetic relationships between alterations in the skirt and LST regions remain unclear. Skirts are identified solely through endoscopic criteria due to a lack of distinct pathological characteristics. Osera and colleagues found that skirts are associated with LST development, with higher incidences of high-grade dysplasia (60.0%) and submucosal invasive cancer (20.0%). However, mutations in commonly varied LST genes (*KRAS*, *PIK3CA*, LOH of 5q) are not detected in skirts [89]. This suggests a different molecular mechanism for lesion development, potentially as a precursor to inflammatory rectal cancer from LSTs.

Similarities and differences between the genetic landscape of LSTs compared to adenoma and polypoid-type carcinoma discussed below are reported in Table 1.

### 3.1. Microsatellite (MSI) and Chromosome Instability (CIN)

Genomic instability plays a critical role in the pathogenesis of CRC during the transition from adenoma to carcinoma, as well as in prognosis and therapeutic strategy. Genome instability is distinguished in microsatellite instability (15%) and chromosomal instability (85%) [90,91]. The most prevalent pathway in LSTs is the CIN, marked by the accumulation of multiple chromosomal aberrations, including gains and losses of DNA, and structural rearrangements, which lead to aneuploidy and widespread copy number alterations [92]. The CIN phenotype is a consequence of cellular replication stress, mitotic errors and telomere dysfunctions, which generates breakage–fusion–bridge cycles, all common events in neoplastic cells. An abnormal number of chromosomes drives a genomic instability that contributes to cancer initiation, progression, and therapeutic resistance [93].

The MSI phenotype represents a distinct form of genomic instability that arises due to defects in the DNA mismatch repair (MMR) system, the guardian of DNA replication fidelity. Persistent errors during this crucial process may lead to the accumulation of insertion or deletion mutations within short, repetitive DNA sequences known as microsatellites. MSI-high (MSI-H) tumors exhibit a high mutational burden, characterized by widespread insertion and deletion mutations, which sustains tumorigenesis and progression of cancer, on one side, but also generates numerous neoantigens, enhancing tumor immunogenicity and responsiveness to immunotherapy [94].

**Figure 3:**
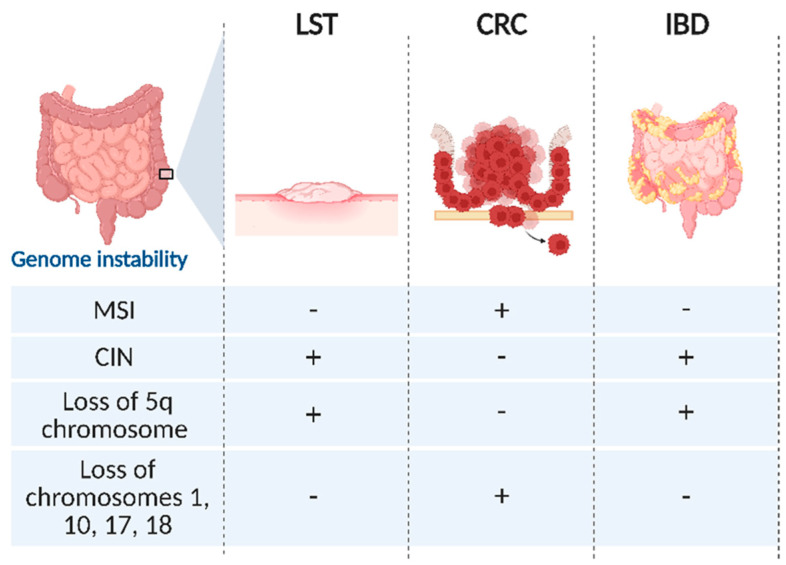
Schematic representation of the genomic instability status of LSTs, compared to CRC and IBD. From a genomic instability perspective, LSTs exhibit a profile that closely resembles that of IBDs with chromosome instability as the prevailing form of genomic alteration and loss of heterozygosity primarily affecting chromosome 5. In contrast, CRC demonstrates a distinct genomic instability pattern characterized by microsatellite instability more frequently observed than CIN. Moreover, LOH events in CRC predominantly involve chromosomes 1, 10, 17, and 18, indicating a broader and more diverse chromosomal involvement compared to LSTs and IBDs. LOH affects mainly chromosomes 1, 10, 17, and 18. CIN: chromosome instability; MSI: microsatellite instability; LOH: loss of heterozygosity.

MSI is notably absent or extremely rare in LSTs, as well as BRAF mutation, which is why no MSI-H cases were detected in the analyzed cohort of LSTs [95] until now. These molecular features allow to distinguish flat lesions from tumors arising via the serrated neoplasia pathway. Conversely, LSTs are more likely to evolve through the CIN way, aligning their molecular profile mainly with precancerous lesions associated with inflammatory bowel diseases (IBDs) (Figure 3).

Additionally, LSTs display specific losses, particularly involving chromosome 5q, overlapping with the inflammatory bowel disease 5 (IBD5) locus implicated in IBD onset [96]. Other losses commonly found in polypoid adenomas, such as chromosomes 1, 10, 17, and 18, containing crucial tumor suppressor genes like *DCC* and *SMAD4*, are rarely detected in LSTs [97,98]. The chromosomal profiles of LSTs differ partially from polypoid adenomas and CRCs, notably featuring chromosome 5q loss, while the hypothetical correlation between LSTs and IBD-associated CRC is worthy to be further investigated.

The similar CIN-driven genomic profile of LSTs and IBD-associated dysplasia may converge on similar mechanisms underlying genomic instability, despite arising in different clinical contexts and having different clinical manifestations.

### 3.2. Genome-Wide Expression Analysis

**Figure 4:**
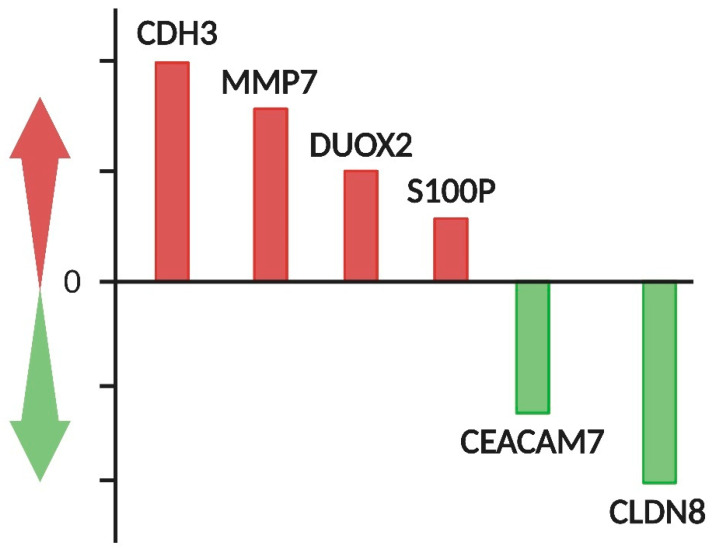
Microarray analysis on LSTs has revealed distinct gene expression patterns when compared to normal colorectal mucosa, highlighting molecular alterations associated with neoplastic transformation. Specifically, four upregulated genes (red bar) and two downregulated genes (green bar), compared to normal mucosa, were detected for the first time in LSTs [99]. Complementing these findings, an independent RNA sequencing (RNA-seq) analysis, conducted on colorectal tumor samples (including LST-adenomas and LST-carcinomas), identified MMP7, DUOX2, CDH3, and CLDN8 aberrantly expressed in LSTs compared to paired normal mucosal tissue [80]. CDH3: cadherin 3; MMP7: matrix metalloproteinase; DUOX2: dual oxidase 2; S100P: S100 calcium-binding protein P; CEACAM: carcinoembryonic antigen related cell adhesion molecule; CLDN8: claudin 8.

Global mutational status and genome-wide expression analyses may unveil transcriptome features involved in LST development. Despite the potentiality of this approach, only a few examples of genome-wide analysis on LSTs are available. For instance, Kita et al. conducted microarray analysis on 12 patients with flat adenomas, revealing the differential expression of 180 genes compared to normal mucosa, with distinct profiles between right and left colon lesions. Few differentially expressed genes were detected, and most of them were correlated for the first time in LSTs (Figure 4) [99].

Similarly, Minemura and colleagues conducted a comprehensive gene expression profile analysis on 41 colorectal tumor samples, including 17 LST-adenomas, 12 LST-carcinomas, and other protruded type tumors. Among the genes significantly upregulated in LST-adenomas, BCL2L1 was meaningfully over-expressed in LSTs [100] and gained attention being implicated in regulating tumor morphogenesis and apoptosis through the control of ROS species and cytochrome *c* release from mitochondria. Conversely to polypoid lesions, the up-regulation of anti-apoptotic genes *Bcl-2* and *survivin* may contribute to LST organization and progression into CRC, by promoting tumor cell survival. *Bcl-2* exhibits heightened expression levels in LSTs, comparable to colorectal adenomas, and functions to inhibit apoptosis, thereby enhancing cell survival. Similarly, survivin, known for its inhibitory effect on apoptosis, has been detected in LSTs [101].

In recent years, advanced technologies like next generation sequencing have significantly advanced our understanding of cancer genetics, including the molecular characterization of LSTs. This progress offers a feasibility for identifying important prognostic and diagnostic molecular markers for detecting pre-cancerous lesions. For example, Hesson and colleagues investigated the mutational profile of low-grade intraepithelial neoplasia, focusing on recurrently mutated genes [80]. In their groundbreaking study, they made the first comprehensive genetic and epigenetic analysis of characteristic molecular pathways in LSTs. They found that mutation rates in LSTs were comparable to microsatellite-stable colorectal cancers (2.4 vs. 2.6 mutations per megabase), with infrequent copy number alterations (averaging only 1.5 per LST). Nong and collaborators further expanded on this work by performing whole-exome sequencing on 14 LSTs with high-grade intraepithelial neoplasia and adjacent non-tumor mucosa revealing a higher molecular burden (3.8 mutations per megabase) than previously reported [102]. These findings elucidate the genomic landscape of LSTs, uncovering frequent alterations in the Hippo/TGF-β and cAMP/Rap1/MAPK pathways. This study also unveils novel insights into the mutational landscape of LSTs, revealing frequent mutations in codon 186 (p.R186C/R186H) of the *GNAS* gene, unique to LSTs and absent in colorectal adenomas, associated with specific tumor locations (rectum) and pathological types (villous adenomas). The *GNAS* gene encodes the G protein subunit *α* (GNAS) which regulates adenylate cyclase activity by coupling with various G-protein coupled receptors. Its involvement in signaling pathways influences cyclic adenosine monophosphate (cAMP) generation, a key mediator in cellular signal transduction and proliferation in certain cell types [103]. The overexpression of GNAS is associated with increased cAMP levels, promoting tumor formation, metastasis, and progression in cancer. While its presence in precancerous lesions like polypoid and serrated-type lesions suggests a role in early tumorigenesis in CRC [104,105], its precise impact on cancer progression from LSTs remains debated.

If chromosomal rearrangements leading to chimeric genes are known in colorectal cancer, the discovery of fusion transcripts in pre-cancerous lesions like LSTs is unexpected. Forty-eight active fusion genes were identified, with the main occurrence in LST-NG type (42 vs. 6 unique fusion genes of LST-G), including hub genes crucial for several cellular processes and potential drug targets. These fusions involve kinases, transcription factors, and affect cellular activities like mRNA stabilization, gene expression regulation, and inflammatory processes (including proteoglycans, insulin signaling, and ErbB signaling). Among all the identified significant genes in this study, authors evidenced an interesting correlation between the up-regulation of NPM1 and YBX1, and their fusion partners, and overall survival of CRC patients. Thus NPM1-PTMA and HIST1H2BO-YBX1 fusion transcripts, found in LST-NGs samples, could be the drivers for progression of flat lesions through cell cycle, proliferation, and apoptosis regulation, and they may also be used as LST prognostic biomarkers [106].

### 3.3. Epigenetic Features of LSTs

Tumor progression results from pathogenic mutations in tumor suppressor genes or the activations of proto-oncogenes, as well as from the accumulation of epigenetic alterations. Among the epigenetic mechanisms, DNA methylation plays one of the main roles in tumorigenesis. Abnormal methylation in promoter regions is the most frequent epigenetic alteration of tumor suppressor gene inactivation. Hence, it is crucial to understand the role of methylated DNA in tumorigenesis [66].

In CRC, methylation markers are categorized into 2 groups. Based on these markers, CRC is classified into high methylation phenotype (or CpG Island Methylator Phenotype, CIMP) which shows the methylation of both groups; intermediate methylation phenotype showing methylation of only group two markers, and low methylation phenotypes showing no marker methylation [107]. Currently, only very few studies have investigated the different expression levels of the methylated phenotype across the whole genome of LSTs.

Based upon a quantitative analysis on the methylation profile of 125 specimens, LSTs were classified into 2 epigenotypes: an intermediate methylation status characteristic of G-LSTs, related to *KRAS* mutations, and a low methylation status specific of NG-LSTs, which correlates with *CTNNB1* mutations. Thus, unlike CRC where methylation phenotypes are classified into high, intermediate, and low groups, LSTs show only intermediate and low methylation phenotypes [66], suggesting a key role of epigenetic aberrations in lesion transformation. Moreover, a significant correlation between the CIMP and LST type was assessed. G-LSTs, particularly those located in the proximal colon, show a high prevalence of CIMP-high and show unmethylation of the mutl homolog 1 (*MLH1*) promoter [108]. On the contrary, serrated adenomas are CIMP-high as well, but they are often associated with methylation of the *MLH1* promoter region [109].

Beyond MLH1, aberrant methylation of both O6-methylguanine-DNA methyltransferase (MGMT) and p16 was also reported in LSTs [110]. The *MGMT* gene encodes a DNA repair protein that removes alkyl groups from the O6 position of guanine to avoid gene mutation and cell death caused by alkylating agents. Alterations in the *MGMT* gene increase the mutation rate and cancer risk and its epigenetic silencing is associated with *KRAS* mutation during colorectal tumorigenesis [111]. Flat-type LSTs also present hypermethylation of the ras association domain family member 1 (*RASSF1*) promoter. Additionally, a correlation between the mutated and methylated genes in this type of advanced adenomas was suggested [112]. In 2017, a whole-genome analysis showed that LST specimens are characterized by a higher degree of methylation status than colorectal polypoid adenomas, but lower than colorectal cancer. In particular, the authors identified positive rates in the methylation levels of two tumor suppressor genes: *RASSF1A*, involved in the cell cycle, apoptosis and genome stability, and Wnt inhibitory factor 1 (*WIF-1*), implicated in Wnt/β-catenin pathway inhibition. In the LST tissues, these two genes were more methylated and transcriptionally downregulated compared to protruded adenomas, but less methylated and more expressed compared to CRC specimens. This finding should be confirmed by increasing the enrolled patients because the cohort was very small [113].

The relevance of unique molecular features of LSTs for predicting their potential malignant progression is evident in the recent case reports of Iwaizumi and colleagues. A woman with more than 150 NG-LST lesions in the large intestine, diagnosed during four years of annual endoscopic surveillance, developed an adenocarcinoma of the sigmoid colon from a single NG-LST. A genomic and epigenomic difference was highlighted by comparing the genetic and epigenetic alterations of adjacent healthy mucosa, four LSTs, and the tumor tissue of the woman. The whole genome analysis allowed the identification of 9 methylated genes in both NG-LST and in adenocarcinoma samples, but none in normal mucosa, with a substantial difference in CpG methylation sites: *ZNF625*, *LONRF2*, *MSC*, *OPLAH* were methylated genes in both NG-LSTs and in cancer, while *SDC2* and *WDR17* were methylated in 3 of the analyzed NG-LST. Conversely, in both pre-cancerous lesions and tumors, the CpG sites of the *PCDHGA4*, *GSG1L*, and *BEND5* genes were hypomethylated [114].

Among the epigenetically regulated genes, identified in this case report, some have been already studied in CRC. *SDC2* encodes syndecan 2, a protein involved in cell proliferation, cell migration, and cell–matrix interactions. Its hypermethylation was also reported by Oh et al. [115]. ZNF625 is a zinc finger protein (ZNFs), probably with a DNA binding transcription factor activity and a role in the regulation of downstream target gene transcription involved in several cellular pathways associated with the onset and progression of colorectal cancer [116]. Lin et al. reported that ZNF625 is one of the most frequently methylated genes in CRC [117]. *LONRF2* codes for the N-terminal domain of LON peptidase and ring finger 2 with a role in protein quality control and its hypermethylation status have also been reported in rectal adenocarcinomas [118]. *WDR17* encodes the WD repeat-containing protein 17; however, this is the first report linking its methylation to colorectal cancer.

Many studies on DNA methylation focus on the impact of this epigenetic mechanism on CpG islands and promoters. Recently, genomic technologies based on sequencing and arrays have allowed the study of methylation at the genomic level and to discover the role of hypermethylated intergenic regions (IGRs), often considered “the dark matter” of the genome. In 2018, Illumina array was applied in the study on the epigenetic differences in LSTs, revealing a decrease in DNA methylation in these “gene desert” regions [119]. The authors showed 1018 IGRs, often annotated as tissue-specific regulatory elements, hypomethylated and epigenetically silenced in normal mucosal tissues. Furthermore, by a comparison of the epigenetic characteristics of colon adenomas, LSTs, and adenocarcinomas, among the IGRs, they identified 435 hypermethylated differentially methylated probes (DMRs) and 417 hypomethylated DMRs. By the analysis of the relative pathways, the authors discovered that genes belonging to four distinct pathways may be the targets of epimutations in LSTs, adenomas, and CRC. Finally, between LSTs and adenomas, the Ras and Raf1 signaling pathway exhibit opposite epimutation patterns with a hypomethylation profile in flat lesions versus a hypermethylation status for adenomas [119].

Finally, Hesson integrated genetic findings with epigenetic and transcriptomic profiles to identify molecular pathways involved in LST development and evolution [80]. Regarding epigenetic mutations, single-molecule bisulfite sequencing and combined bisulfite restriction analysis (COBRA) detected 50% methylation in 13 out of 17 genes studied in a cohort of 11 LSTs. In this study, the authors demonstrated that epigenetic inactivation is more common in G-LSTs than in NG-LSTs. Interestingly, among the genes, the most frequently epigenetically inactivated are those involved in axonal guidance such as *SLITRK2*, *SLITRK5* (methylated in 98% of cases), as well as *ANO5* (methylated in 63% of cases). Being also frequently mutated, axonal guidance signaling seems a genetic and epigenetic target for both LSTs and CRC.

Several studies, focused on the methylation profile of CpG islands in LSTs, have evidenced significant epigenetic differences between LSTs, adenomas, and CRCs, even if currently their involvement in neoplastic transformation and progression is unknown. Variation in the degree of methylation, as well as the genes and pathways involved, allows distinguishing G-LSTs and NG-LSTs on the basis of molecular features. Further exploration about the interplay between genetic and epigenetic alterations across various LST subtypes is warranted to deepen our understanding of their evolution in neoplasia.

## 4. Conclusions and Future Perspectives

Until research under “omic” sciences became more widespread, precancerous lesions, known as laterally spreading tumors for their morphology, were prevalently detected and studied in Asian countries and their categorization into granular and non-granular was based on morphology. Notwithstanding, thanks to recent advancements in endoscopic techniques, LSTs have been discovered with major frequency, thus attracting the attention of the international scientific community for a better classification and comprehension of the mechanisms of their transformation in neoplastic lesions. Overall, while LSTs are not classified as malignant tumors, they have an intrinsic potential to evolve into CRC via dysplastic progression, accumulation of genetic/epigenetic mutations, and microenvironmental influences. The evidence suggests that LSTs display a peculiar mutational landscape. Intriguingly, the present review highlighted that LSTs are characterized by peculiar molecular alterations, such as axon guidance pathway dysregulation or the fusion genes HIST1H2BO-YBX1. In LSTs, their dysregulation may explain an early molecular divergence from conventional adenomas, likely driven by chromosomal instability, and distinct oncogenic pathways which trigger their transformation. Such divergence could underly the unique non-polypoid, laterally expanding growth pattern observed in LSTs. Clinically, these novel findings could help in distinguishing LSTs from polypoid lesions, especially in ambiguous histological cases, and in better correlating the endoscopic appearance of flat lesions with their invasiveness or recurrence risk. Indeed, morphological features alone remain insufficient to reliably predict the invasive behavior of non-polypoid lesions. Thereby, an integrative “omic” approach, especially transcriptomic, epigenomic, and proteomic profiling, hold immense potential in pinpointing robust diagnostic and prognostic biomarkers. The differentiation of benign LSTs from those with malignant potential has huge clinical implications because it may support minimally invasive endoscopic resection. The identification of specific molecular alterations (e.g., in Wnt, actin cytoskeleton, and axon guidance pathways) could allow a better prediction of the malignant potential of LSTs, which is crucial for determining surveillance intervals and management strategies, especially in patients with IBD-associated dysplasia.

LSTs’ molecular features show substantial overlap with those observed in IBD-related neoplasia suggesting common pathways of chronic inflammation-induced tumorigenesis. The integration of the molecular profiling of flat precancerous lesions into clinical practice holds significant promise for enhancing risk stratification and the personalized management of LST and IBD patients. Specifically, surveillance strategies could be refined by including molecular markers alongside endoscopic criteria, such as lesion morphology and histopathology. Furthermore, treatment decisions may be better guided toward a personalized therapy based on the identification of patients at higher risk of progression. This aspect is particularly relevant for patients with IBD-associated CRC, for whom chronic inflammation and immunosuppressive therapy complicate the standard treatment approach.

This review lays the ground for an urgent need to elucidate the molecular landscape of LSTs, which follow distinct tumorigenic pathways compared to conventional polyposis and serrated lesions, meanwhile, they share pathways of inflammation and neoplasia with other bowel diseases. The convergence of molecular pathology and clinical practice highlights the need for multidisciplinary research to ultimately acquire further knowledge for the development of precision strategies for early detection and prevention, contributing to improving management and outcome in both sporadic and IBD-associated colorectal cancer.

## Figures and Tables

**Table 1:** Genetic landscape of LSTs.

Mutation	Pathway/Function	LST	Adenoma/Polypoid Carcinoma	Correlation	References
*KRAS*	MAPK signaling pathways	G-LSTs in distal colon	More frequent in early-stage carcinomas and protruded type adenomas	COX-2 overexpression Gastrin overexpression	[27,67,68,72,76,87]
*APC* (5q chromosome)	Wnt signaling pathway	LOH at 5q chromosome in NG-LTSs	Loss of function of APC	High levels of CCND1 and c-Myc Nuclear β-catenin accumulation β-catenin resistant to ubiquitination Adherence proteins expression Missense mutation of MED12L	[22,76]
β-catenin		High expression Low level of mutation of *CTNNB1* in NG-LSTs	Low expression High levels of *CTNNB1* mutations		[22,37,38,42,56]
*TP53* (17p chromosome)	DNA repair/genome stability	Mostly in NG-LSTs	Undetected in adenomas, but detected in cancer derived from malignant progression of LSTs LOH 17p chromosome is crucial in progression from adenoma to cancer		[56,69,71,76]
*PIK3CA*	AKT signaling pathway	LSTs with high pathological grades Mostly in G-LSTs	Present in CRC (10–30%), but not detected in polypoid/depressed and flat-elevated adenomas	*KRAS* mutation	[88]
*BRAF*	RAS/RAF/MEK/MAPK signaling pathway	Rare in LSTs	Rare in CRC	Mutually exclusive with *KRAS* mutation Concurrent mutation with *KRAS*	[76]
*SLITRK*, *SLIT*, *NTRK* genes family	Axonal guidance pathway	Frequently mutated	Not in polypoid adenomas, but mutated in CRC		[80]

Abbreviations: *APC*: adenomatous polyposis coli; *BRAF*: B-raf; CCND1: cyclin D1; COX-2: ciclooxigenase 2; CRC: colorectal cancer; *CTNNB1*: catenin beta 1; G-LSTs: granular LSTs; *KRAS*: activating Kirsten rat sarcoma virus; LOH: loss of heterozygosity; LSTs: laterally spreading tumors; MAPK: of mitogen-activated protein kinases; MED12L: mediator complex subunit 12L; NG-LSTs: nongranular LSTs; *PIK3CA*: phosphatidylinositol-4,5-bisphosphate 3-kinase catalytic subunit alpha; *TP53*: tumor protein p53.

## Abbreviations

The following abbreviations are used in this manuscript:

CIMP	CpG Island Methylator Phenotype
CIN	Chromosomal instability
COBRA	COmbined Bisulfite Restriction Analysis
CRC	Colorectal Cancer
DMRs	Differentially methylated probes
ESGE	European Society Gastrointestinal Endoscopy
G-H-LST	Granular homogeneous LST
G-LST	Granular LST
G-M-LST	Granular mixed LST
IBDs	Inflammatory Bowel Diseases
IGRs	Intergenic regions
LOH	Loss of heterozygosity
LSTs	Laterally Spreading Tumors
MMR	Mismatch repair
MSI	Microsatellite instability
MSI-H	Microsatellite instability-High
NG-LST	Nongranular LST
NG-F-LST	Nongranular flat-elevated LST
NG-PD-LST	Nongranular pseudo-depressed LST
RNA-Seq	RNA sequencing
SMI	Submucosal invasion
